# Mammoth Bilateral Ovarian Serous Cyst Adenomas in a Postmenopausal Woman: A Rare Case Report

**DOI:** 10.7759/cureus.48935

**Published:** 2023-11-17

**Authors:** Ivan Novakov, Pavel Timonov, Antoaneta Fasova

**Affiliations:** 1 Special Surgery, Medical University, Plovdiv, BGR; 2 Forensic Medicine, University Hospital St. George, Plovdiv, BGR; 3 Forensic Medicine and Deontology, Medical University, Plovdiv, BGR; 4 Anatomy, Histology and Embryology, Medical University, Plovdiv, BGR

**Keywords:** hysterectomy, ovarian cysts, postmenopausal woman, laparotomy, mammoth ovarian serous cyst-adenoma

## Abstract

Serous cyst adenomas account for about 40% of all ovarian tumors and most commonly are diagnosed in middle-aged women. The aim of this publication is to present an extremely rare case of mammoth bilateral cyst adenomas in a postmenopausal woman, occupying the pelvis and the most of abdominal cavity.

A 67-year-old obese woman was presented to our emergency department with abdominal pain. Ultrasound and computed tomography of the abdomen verified two huge cystic masses. Exploration of the abdominal cavity by laparotomy established two intact giant cystic masses with ovarian origin. The cysts were removed by bilateral salpingo-oophorectomy and hysterectomy was performed. Histological examination revealed that both cystic masses were ovarian serous cyst adenomas. The woman was discharged with an uneventful recovery. We present a case of the largest gigantic bilateral ovarian cyst adenomas in the oldest woman ever reported.

## Introduction

Ovarian cysts more than 10 cm are considered “large ovarian cysts”. The two most common variants of large ovarian cysts are mucinous and serous cyst adenomas. Serous cyst adenomas account for about 40% of all ovarian tumors and most commonly are diagnosed in middle-aged women. In more than 10% of cases, these tumors are bilateral [1-4].

While the growth of mucinous cyst adenomas is not so rare to occupy the abdominal cavity, serous cyst adenomas are smaller in their size. The aim of this publication is to present an extremely rare case of mammoth bilateral cystadenomas in a postmenopausal woman, occupying the pelvis and the most of abdominal cavity.

## Case presentation

A 67-year-old obese woman was presented to our emergency department with weakness, nausea, dyspnea, obstipation, and mild abdominal pain. Although had noticed distension in her abdomen for several months no medical advice was looked for. No history of any surgical interventions was reported.

Due to the vigorous weakness and being severely obese with a body mass index >40.0 physical examination was possible only in the lying position of the woman on her left side. Abdominal examination revealed a distended abdomen with asymmetry (more prominent on the left flank), a slightly tender abdominal wall with a huge palpable mass, and diminished intestinal peristaltic sounds.

**Figure 1:**
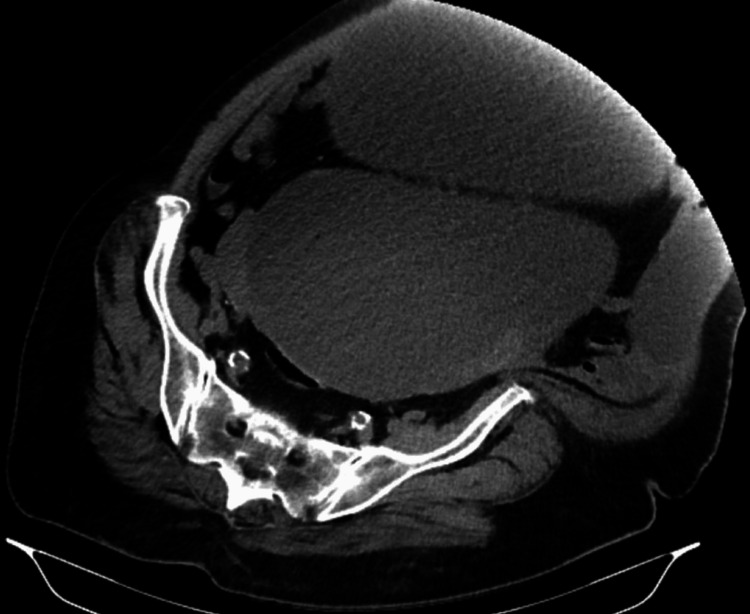
CT scan of the cyst.

Abdominal ultrasound verified a large cystic mass, but due to the extreme obesity and lying in the lateral position of the woman no detailed information was obtained. Computed tomography of the abdomen and pelvis, which was performed after hospitalization of the women revealed two giant cystic masses: one with a transverse dimension of 27 cm and sagittal of 33 cm, the second 17 x 20 cm, respectively, both with a density up to 18 Hounsfield units (Figure 1). The serum level of tumor marker CA 125 was 198.4 U/mL (0-35 U/mL). 

**Figure 2:**
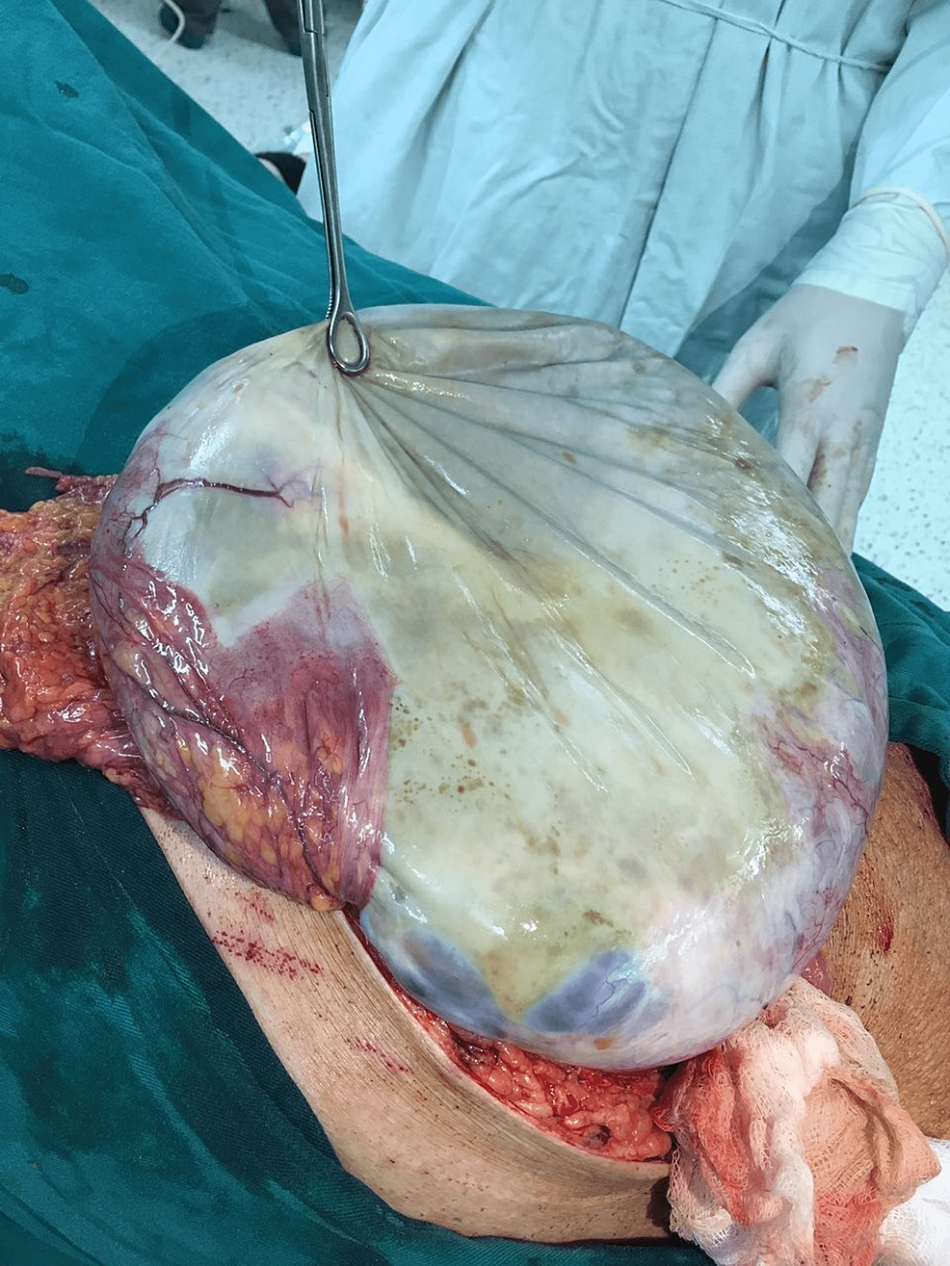
Gross pathology presentation of a mammoth right ovarian cystic mass.

**Figure 3:**
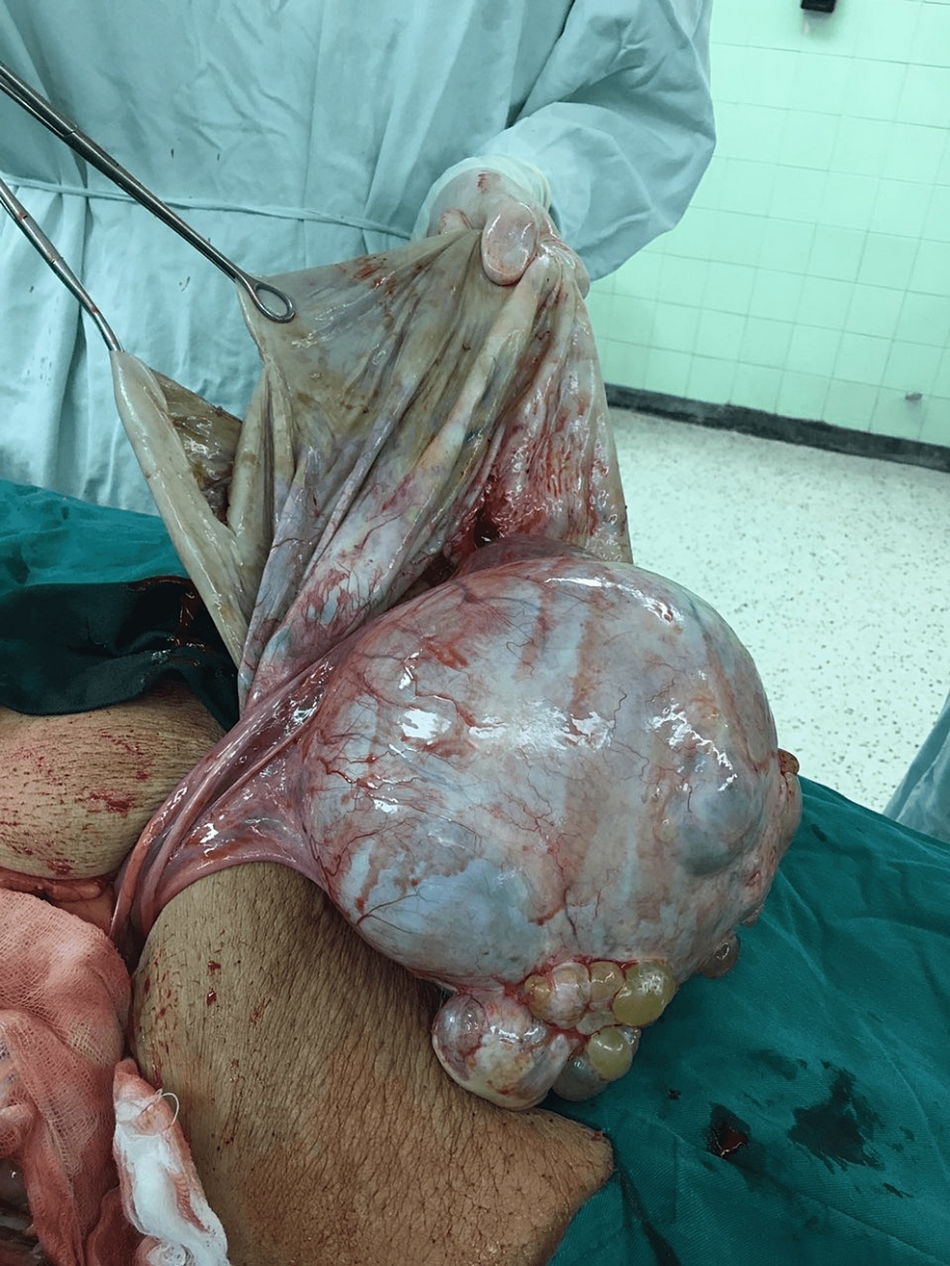
The right ovarian cystic mass after partial aspiration of its liquid content.

On the second day of her admission, the woman underwent a laparotomy. Because the woman was too obese to fit on the operating table in the supine position she was placed in the left lateral position and the abdomen was opened by the right paramedian incision. Exploration of the abdominal cavity revealed two intact giant cystic masses with smooth shiny outer surfaces and adherent great omentum to them. The first one of the cysts was more than 60 cm in its greatest diameter (Figure 2). The cyst was incised with slow aspiration of its liquid content to prevent acute reduction of the abdominal cavity pressure with caution not to disseminate the content of the cyst to the peritoneal cavity considering the high serum level of CA 125. Multiloculality, with the origin of the cyst from the left ovary, was established (Figure 3). After cutting the adhesions between the omentum, the cyst was removed from the peritoneal cavity.

**Figure 4:**
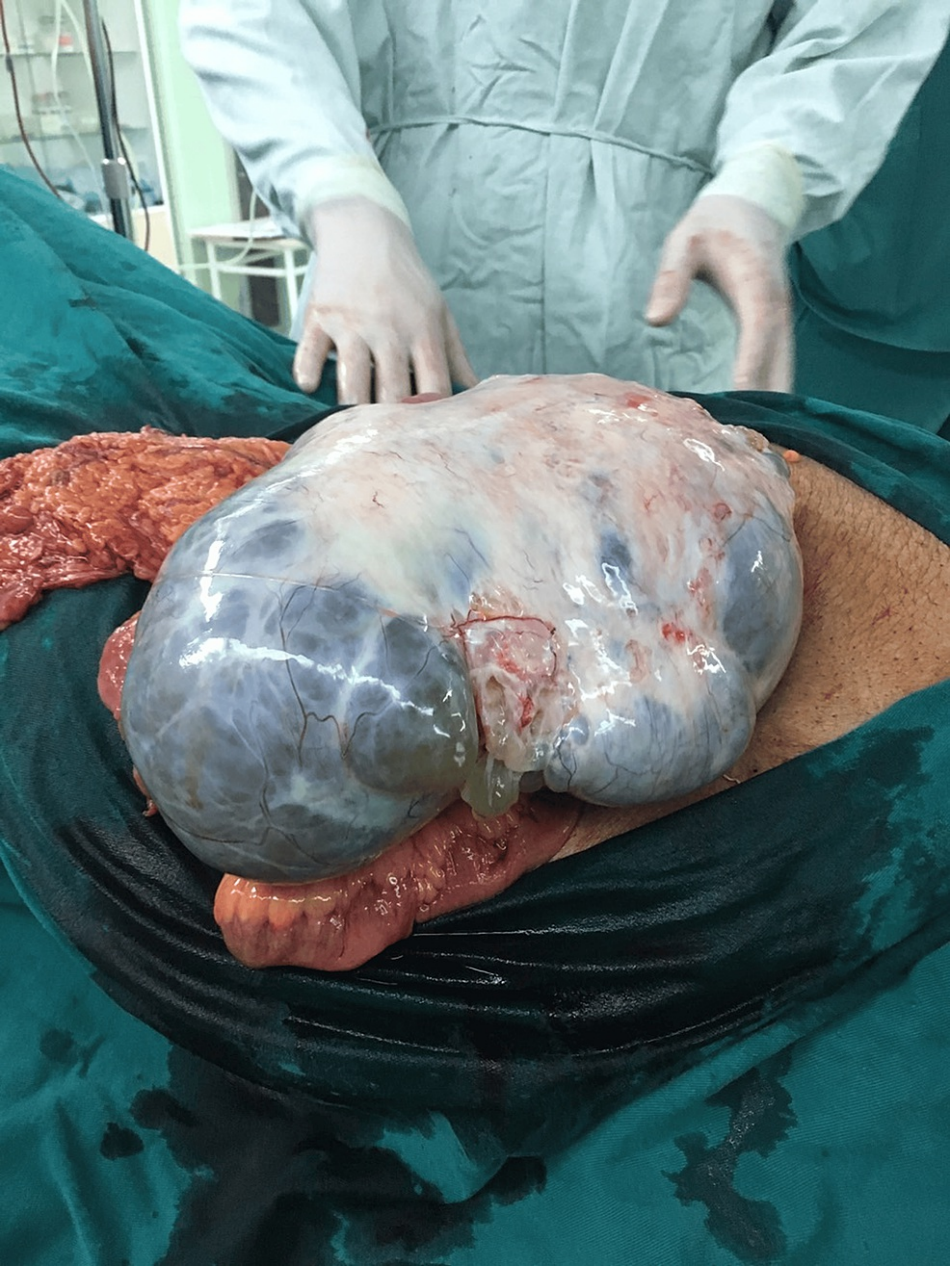
Gross picture of intact multilocular left ovarian cystic mass.

**Figure 5:**
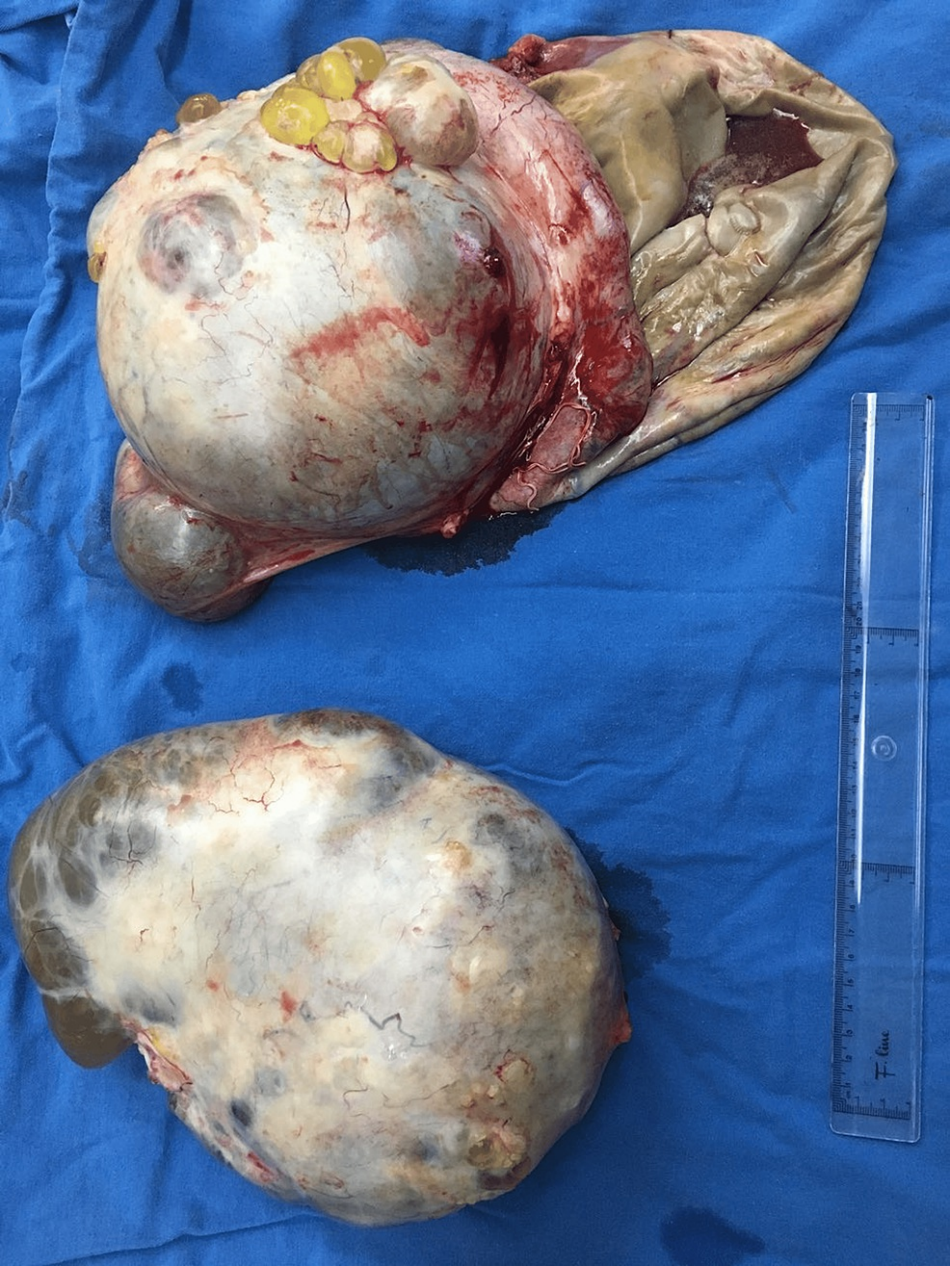
Macroscopic appearance of both cystic masses after their removal from the peritoneal cavity.

The second cyst was also multilocular, with the greatest diameter of more than 40 cm (Figure 4). This cyst was removed intact (Figure 5). It was established that the origin of the cysts is the right ovary.

After removing the cysts by bilateral salpingo-oophorectomy, a hysterectomy was done. Two tube drains were placed into the pelvis and the abdominal cavity was closed. Low molecular weight heparin against thromboembolism was administered after surgery. The woman was discharged on the 11th postoperative day with an uneventful recovery.

Histological examination revealed that both cystic masses were benign ovarian serous cyst adenomas, with no other pathology of the ovaries, tubes, and uterus. Four years later during the accidental visit of the woman, it was established that obesity was persistent and no abdominal complaints. Written informed consent was obtained from the patient for his anonymized information to be published.

## Discussion

Serous cyst adenomas are benign tumors that arise from the surface epithelium of the ovary. These tumors are quite common and they mostly occur in the third, fourth, and fifth decades of life. Serous cyst adenomas vary in size from a few centimeters to huge ovarian cysts [1-4]. Although not so rare, these tumors can be presented as “large ovarian cysts.” Abundant secretion and extreme growth with occupation of the abdominal cavity are not so typical for them [5-8]. Publications of mammoth ovarian serous cyst adenomas in medical literature are very limited. We established only a few cases of enormous ovarian serous cyst adenomas occupying the most of abdominal cavity available. In all these published cases the adenomas are unilateral, settled in women up to 30 years [1-8]. With this publication, we aim to report a unique case of bilateral mammoth ovarian serous cyst adenomas in a postmenopausal woman, which occupied the peritoneal cavity.

The giant ovarian cysts are considered chronic causes of elevated intra-abdominal pressure [9]. Elevated diaphragm, reduction in compliance of the lung, chest, and abdominal wall, diminished venous return, and celiac blood flow are pathophysiologic changes in the state of intra-abdominal hypertension that are manifested with cardiovascular, pulmonary, and gastrointestinal symptoms [9]. We consider that in our case weakness, nausea, dyspnea, and abdominal pain are signs of intra-abdominal hypertension.

Removing the giant ovarian cyst is associated with the risk of intra- and postoperative morbidity. Factors that influence this morbidity are duration of operation, technically difficult surgery, abundant blood loss, and sudden reduction of intra-abdominal pressure. Sudden reduction of intra-abdominal pressure is the main operative and postoperative problem in women with excision of giant ovarian cysts. After sudden abdominal decompression, there is a potential risk of cardiorespiratory failure due to the paradoxical movement of the thinned diaphragm, laxity of the abdominal wall, changes in abdominal vascular tone, and postoperative pain. Altered mechanics of ventilation may cause life-threatening pulmonary edema [9,10]. Additionally, after removing the giant ovarian cysts there is a risk of fatal intestinal distension due to the placement of the intestine in a redundant abdominal cavity. However, in the presented case of the obese postmenopausal woman with removed two mammoth ovarian cysts from the peritoneal cavity postoperative period was uneventful.

The raised serum level of CA 125 that was established at the admission of the woman was suggestive of ovarian cyst adenocarcinomas. However, CA 125 is not tumor tumor-specific antigen. We consider the presenting woman as one of the 1% group of “normal women” with the rising of this tumor marker [11]. The benign origin of both cystic masses was verified histologically and the good condition of the woman after four years is confirmation that the elevated serum level of CA 125 was not connected with ovarian malignancy. Additionally, peritoneal irritation by these huge cystic masses is one more potential cause of elevated serum levels of CA125 observed.

## Conclusions

Our case is unique because it presents the largest gigantic bilateral ovarian cyst adenomas occupying the abdominal cavity in the oldest woman ever reported. Additionally, it presents those extremely rare cases of “normal women” with rising CA125 without malignancy. We consider this publication might be useful for practice.

This case report emphasizes the significance of a thorough evaluation of all women presented with abdominal pain. Although the condition is common, it is potentially dangerous in its massive form if not timely diagnosed and managed properly.
